# Sir Roy Calne: a renaissance man of modern medicine

**DOI:** 10.3389/frtra.2025.1765992

**Published:** 2026-01-27

**Authors:** Angeles Baquerizo, Neville Jamieson, Peter Friend

**Affiliations:** 1 Terasaki Foundation Laboratory, Terasaki Institute for Biomedical Innovation, Los Angeles, CA, United States; 2 Clinical Professor Surgery, Scripps Center for Cell & Organ Transplantation, La Jolla, CA, United States; 3 Department of Surgery, University of Cambridge, Cambridge, United Kingdom; 4 Department of Surgical Sciences, University of Oxford Nuffield, Oxford, United Kingdom

**Keywords:** art, immunosuppression, leadership, surgery, transplantation, research

## Abstract

It is a challenging task to encapsulate the prolific life and accomplishments of Sir Roy Calne, a unique individual and pioneer who transformed the impossible into clinical reality, forging a new era of organ transplantation. In the annals of medical history, few figures embody the intersection of science and humanity as did Sir Roy Calne. A pioneering transplant surgeon, scientist, accomplished artist, visionary leader, and devoted family man, Calne's multifaceted legacy continues to influence both medical science and the arts, demonstrating how these two fields can intersect to advance human understanding and compassion. Sir Roy Calne died peacefully in Cambridge on January 6, 2024, leaving behind a legacy that continues to define the field of transplantation.

## Early life and education

Sir Roy Yorke Calne, born on December 30, 1930, in Richmond, Surrey, into a family with a passion for engineering, Sir Roy Calne's early life was steeped in a culture of innovation. His father, a car engineer, and his mother, Eileen, instilled in him the values of perseverance and curiosity.

He was educated at Lancing College and went on to train as a doctor at Guy's Hospital, London, beginning at the age of 16, and completed his medical education in 1952. At an early stage he became aware of the plight of young patients with kidney failure and the potential benefits that would be delivered if organs could be transplanted successfully. He learned of the seemingly impossible problem of rejection and committed himself to finding a solution. His early kidney transplant experimental work at the Royal College of Surgeons of England led to a Fellowship in the laboratory of Joe Murray in Boston. He returned to a staff position at Westminster Hospital, London, and was appointed at the age of 35 years to the Chair of Surgery at the University of Cambridge in 1965, where he developed a highly successful, academically based multi-organ transplant program (1). He retired from the Chair in 1998.

## The surgeon-scientist

Calne's career was marked by a series of ground-breaking achievements which straddled the divide between the technical aspects of transplant surgery and the science of transplant immunology. In 1968, he performed the first liver transplant in Europe, a monumental achievement that defied considerable opposition. He dedicated himself over the next 15 years to transforming what was believed by many of his peers to be a fruitless endeavor into a highly effective therapy for patients with end-stage liver failure. His persistence (and that of his co-pioneer and friend Thomas Starzl) was rewarded by the progressive acceptance and adoption of liver transplantation worldwide from the mid-1980s.

In 1987, he was part of the team that performed the world's first liver, heart, and lung transplantation. His relentless pursuit of medical innovation also led to the first intestinal transplant in the UK in 1992 and the first successful combined stomach, intestine, pancreas, liver, and kidney cluster transplantation in 1994. Calne's wide-ranging interests included his early work in xenotransplantation, which he explored as a potential solution to the shortage of human donor organs.

Despite his extraordinary contributions to the surgical aspects of transplantation, Calne's greatest impact resulted from his work in immunosuppression. In 1960, he demonstrated that 6-mercaptopurine (6-MP) could prolong the survival of transplanted kidneys in the dog. This led to the use of azathioprine, which proved more effective and, in combination with steroids, provided the mainstay of clinical immunosuppression until the 1980s. His experiments with the immunosuppressant ciclosporin A in the late enabled the successful establishment of liver, heart and lung transplant programs around the world, by dramatically raising the 1-year graft survival rate from under 30% to over 70%. Later vital contributions to immunosuppression included early experimental and clinical work on FK506 (tacrolimus), Campath (alemtuzumab), and rapamycin (sirolimus).

Beyond his technical and clinical contributions, Calne's influence extended to his role as a mentor and educator. Through his work as a surgeon at Addenbrooke's Hospital in Cambridge, as the Professor of Surgery at the University of Cambridge, and as a Fellow of Trinity Hall, he trained and inspired medical professionals from across the globe. Many of these individuals went on to establish their own successful programs in their home countries and continued to advance the field of transplantation (2).

## Awards and honors

In recognition of his contributions to medicine, Calne received numerous awards. He was elected Fellow of the Royal Society in 1974 and was knighted in 1986. His achievements were further honored with the Lister Medal in 1984, for which he delivered the Lister Oration in May 1985, titled “Organ transplantation: from laboratory to clinic”, the Cameron Prize for Therapeutics of the University of Edinburgh in 1990, the Pride of Britain Lifetime Achievement Award in 2014, and shared the Lasker-DeBakey Clinical Medical Research Award with Thomas Starzl in 2012. He also received the prestigious Medawar Prize from The Transplantation Society in 1991, underscoring his global impact on transplantation medicine.

## The artist's eye and humanitarian leader

**Figure 1:**
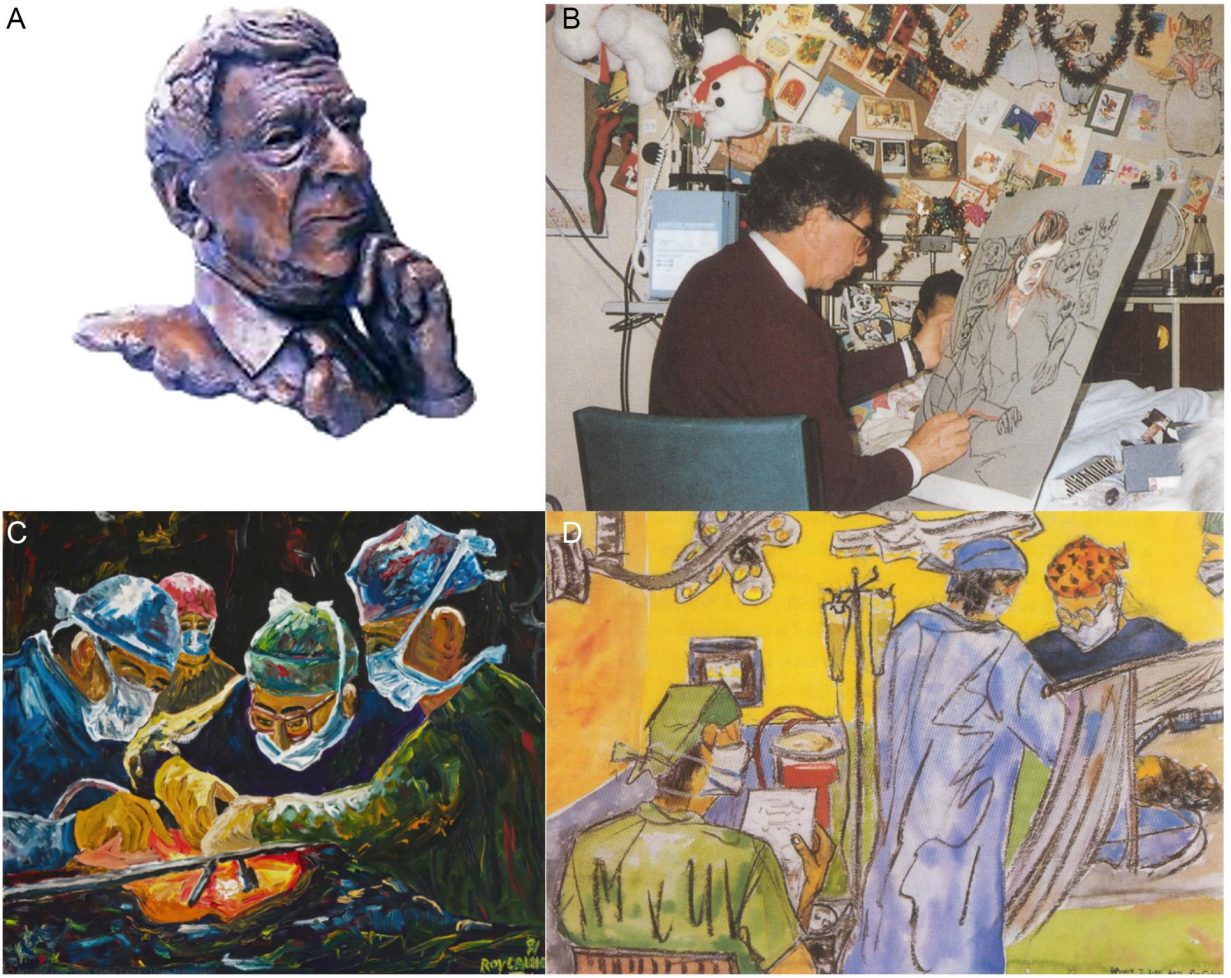
**(A)** A bronze bust of Sir Roy Calne by sculptor Laurence Brederick, outside the main operating theatres at Addenbrooke's hospital. **(B)** Calne sketching a child at Addenbrooke's hospital 1991. **(C)** Liver transplant, 1991 by Roy Y. Calne. **(D)** “Heart, lungs and liver transplant in progress at Papworth Hospital”, sketched by R.Y. Calne in the operating room, March 7th, 1991 at 4.00 am.

Calne's approach to medical advance was characterized by a fundamental interest in and understanding of the underlying science; he combined this with creative thinking, qualities that would later manifest in his artistic endeavors. He demonstrated that technical excellence and human compassion need not be mutually exclusive – indeed, they can reinforce each other in powerful ways. His integrity and genuine care for his patients infused everything he did, from pioneering surgical techniques to capturing the essence of his patients' experience in their portraits (Figure 1).

What set Calne apart was this unique ability to bridge the gap between medicine and art. Initially an amateur artist, he found inspiration in the Scottish painter John Bellany, particularly after Bellany's liver transplant in Cambridge in 1988. Bellany's determination to paint immediately after his surgery deeply moved Calne, inspiring him to develop his own artistic practice. His artistic style, characterized by expressive brushwork and vivid colors reminiscent of German Expressionism, captured the raw emotional and physical experiences of transplant patients and medical staff alike. What he lacked in formal artistic training, he made up for with profound emotional authenticity and intimate knowledge of his subjects.

The culmination of this artistic journey was “The Gift of Life”, an exhibition of paintings by Professor Sir Roy Calne promoting organ donation awareness, which opened at London's Barbican Centre in 1990. Curated by John Sheeran and Imogen Lock, the exhibition featured Calne's portraits of transplant patients, scenes from operating theaters, and studies of medical staff. Particularly moving were his depictions of intensive care nurses, capturing the dedication of these essential healthcare workers. His paintings didn't just document medical procedures; they captured the hope, fear, courage, and resilience of patients, as well as the compassion of healthcare professionals.

Calne's approach was marked by a rare combination of vision and empathy. He also understood that advancing medical science required not just technical expertise but also public understanding and support. This insight led him to use his art as a tool for advocacy, particularly in promoting organ donation awareness.

The impact of this approach became evident as “The Gift of Life” toured internationally, reaching audiences in the United States, Singapore, and Japan. In Japan, where first legal and later cultural barriers had limited access to organ transplantation, Calne's artistic depictions helped facilitate important discussions about organ donation and medical ethics. As John Sheeran reflected on Calne's “The Gift of Life” exhibit, “Roy possessed something almost saintly. He was an amateur artist, but it was the feelings that his paintings expressed about his patients and the medical teams that made such an impact with the public. It was Roy's humanity that shone through; he cared enough about them all to express what he thought and felt. His paintings gave an immediately accessible human dimension to a challenging subject, touching people's hearts as well as their minds” (3).

## As a leader and family man

**Figure 2:**
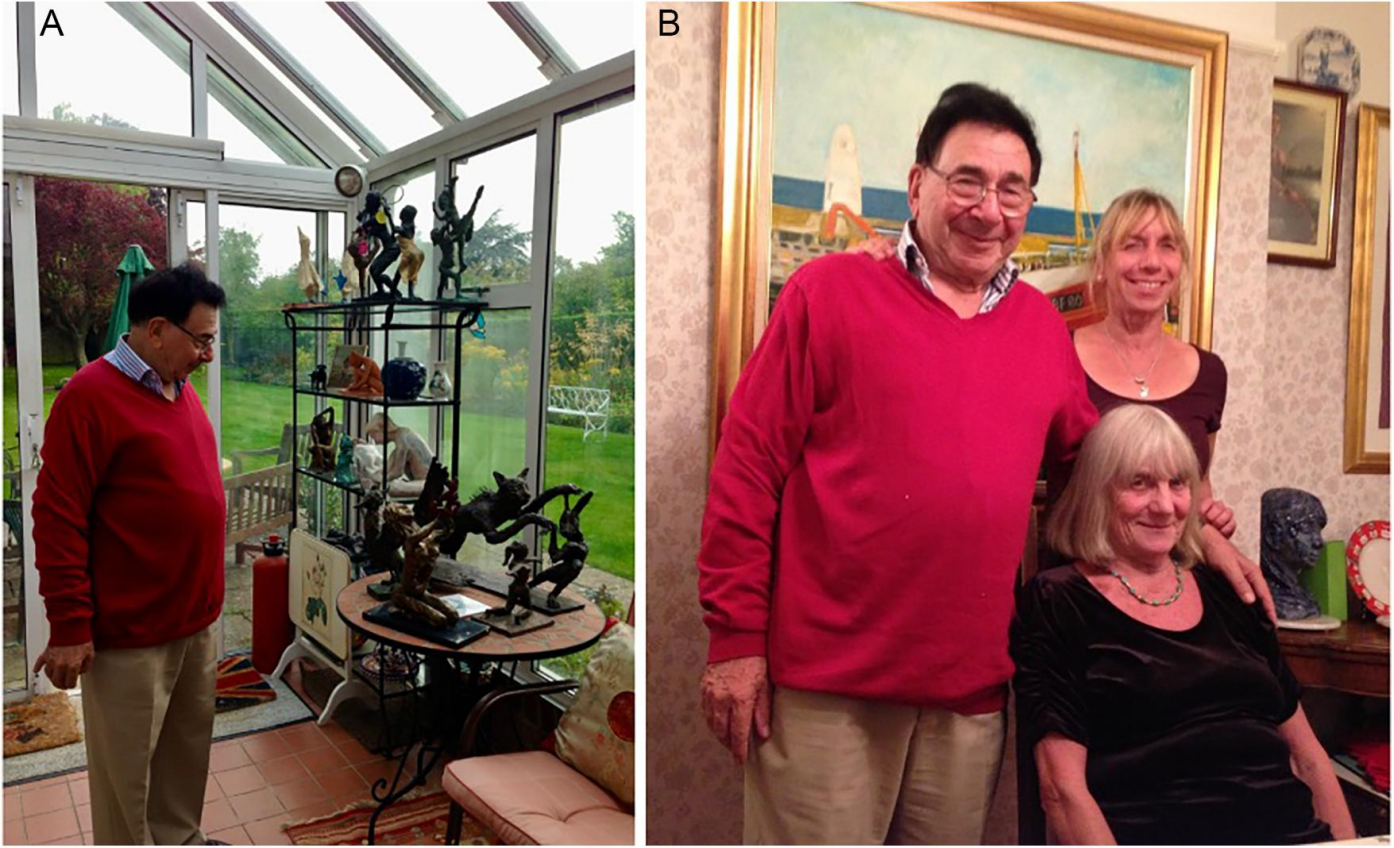
Dinner at Calne's home (September 25th, 2013). (A) Showing his sculptures (B) With his wife Patsy and daughter Jane.

Behind the accolades and achievements, Calne was also a devoted family man. His wife Patsy was not just a life partner but an intrinsic part of the transplant enterprise: their partnership exemplified the dedication required in pioneering medical work. Members of the Cambridge transplant unit, both surgical and scientific, were adopted as part of an extended family, providing a warm welcome to staff and visitors from around the world. The Calne household was known for its hospitality, regularly hosting gatherings that brought together family, colleagues, and friends in a celebration of both professional achievement and personal connection. His daughter Suzanne followed in his footsteps as an ICU nurse, and Jane as a family doctor, demonstrating how the values of compassion and service were passed down through generations (Figure 2).

## Roy Calne's legacy

Sir Roy Calne's legacy is unique in modern medicine. He demonstrated a combination of scientific and surgical innovation that truly transformed his field. He did so with resources that were very limited by today's standards: his achievements required an astonishing degree of determination - willingness not only to attempt the near-impossible, but also the conviction that, even in the face of initial setbacks, the goal could be reached. While his scientific and surgical achievements stand as landmarks in transplant surgery, his ability to humanize the field through art further sets him apart. In an era of increasing medical specialization and technological advancement, Sir Roy Calne's example reminds us that the very best of healthcare combines scientific rigor with humanity. His legacy continues to inspire not just through the lives he saved directly, but by his example of what can be achieved by vision, resilience and compassion, and - his unique approach to the practice of medicine.

As we remember Sir Roy Calne, we celebrate a life devoted to the betterment of humanity through science and compassion.

## Data Availability

The raw data supporting the conclusions of this article will be made available by the authors, without undue reservation.
